# Antioxidant Potential of Polyphenols and Tannins from Burs of *Castanea mollissima* Blume

**DOI:** 10.3390/molecules16108590

**Published:** 2011-10-12

**Authors:** Shan Zhao, Jie Yuan Liu, Si Yu Chen, Ling Ling Shi, Yu Jun Liu, Chao Ma

**Affiliations:** National Engineering Laboratory for Tree Breeding, College of Biological Sciences and Biotechnology, Beijing Forestry University, Beijing 100083, China

**Keywords:** *Castanea mollissima*, polyphenols, tannins, antioxidant, chestnut burs

## Abstract

Spiny burs of *Castanea mollissima* Blume (Chinese chestnut) are usually discarded as industrial waste during post-harvesting processing. The objective of this study was to establish an extraction and isolation procedure for tannins from chestnut burs, and to assess their potential antioxidant activity. Aqueous ethanol solution was used as extraction solvent, and HPD 100 macroporous resin column was applied for isolation. The influence of solvent concentration in the extraction and elution process on extraction yield, tannins and polyphenols content, as well as antioxidant potential, including DPPH and ABTS radical scavenging ability, reducing power ability and cellular antioxidant ability were assessed. In both the extraction and isolation process, 50% aqueous ethanol led to superior total tannins and polyphenols content as well as significantly higher antioxidant activity. In addition, the antioxidant activity and the total tannins content in extracts and fractions had a positive linear correlation, and the predominant components responsible for antioxidant activities were characterized as hydrolysable tannins. To the best of our knowledge, this is the first report on the enrichment of tannins from burs of *C. mollissim* using macroporous resin chromatography, and to assess the cellular antioxidant activity of them.

## 1. Introduction

Chinese chestnut (*Castanea mollissima* Blume) belongs to the Castanea family and is a wood plant widely cultivated in Europe, North American and Asia as an economic crop. Its sweet kernels are edible and have been used in traditional Chinese medicine for treatment of gastroenteritis, bronchitis, and regurgitation [1]. In China, Chinese chestnuts are widely cultivated in Hebei, Shandong, Hunan, Jiangxi, Fujian and Anhui provinces, with a total annual kernel production of up to 460,000 t, contributing to approximately 60% of the global production. Chestnut burs with 1 to 2 cm long, 1 mm thick spines, representing about 20% of the total chestnut dry weight, are byproducts of chestnut cultivation, usually being discarded upon harvesting. Therefore, the use of chestnut burs as potential source of natural antioxidants and functional food ingredients is of great interest to the chestnut industries.

Over the past few years, much attention has been paid to natural polyphenols such as silymarin, curcumin, green tea, and grape seed extracts [2], which could provide protection against the harmful effects of free radicals and reduce the risk of several diseases, including cancer [3,4] and inflammation [5], as well as to modulate immune responsiveness [6]. Although some papers have been published regarding the antioxidant activity and phenolic constituents of chestnut seed shells [7,8] and kernels [9], little is known about the constituents and bioactivities of Chinese chestnuts burs. Recently, Vázquez and co-workers [10,11] did lots of valuable studies on the polyphenols from burs of *C. sativa*. They found that the extract from *C. sativa* burs exhibited antioxidant potential in the DPPH (1,1-diphenyl-2-picrylhydrazyl), ABTS^+^ (2,2′-azinobis-3-ethylbenzothiazoline-6-sulfonic acid) radicals and reducing power assays. Mujić and co-workers also reported that the spiny burs extracts of *C. sativa* could increase rat pancreatic β-cell viability after streptozotocin treatment by protecting DNA from oxidative damage and by enhancing the natural antioxidant system [12]. To obtain the polyphenols from the burs of *C. sativa*., Vázquez tried several extraction solvents, and found that the extraction yield, the total polyphenols content and antioxidant activity of extracts decreased with the polarity of the solvent [10], and recommended ethanol as the extraction solution for food or pharmaceutical application due to its GRAS (General Recognized as Safe) status [11]. However, the polyphenols content of crude extract was only 26.32 g gallic acid equivalent (GAE)/100 g extract, which was not good enough for further studies and industrial applications. Therefore, there was still an urgent need to establish an efficient and low cost method to enrich the polyphenols and tannins in extract. Thus, the objective of this study was to establish such a procedure for extraction and isolation, as well as to assess the antioxidant activity of the resulting extracts.

## 2. Results and Discussion

### 2.1. Extraction of Polyphenols and Tannins

The polyphenols and tannins contents of different extracts are presented in Table 1. The data therein shows that the yield of extract decreased significantly with increasing ethanol concentration. The extraction with water led to the highest extraction yield, followed by 30% ethanol, 50% ethanol and 75% ethanol. This trend was consistent with the report of Vázquez [10], who measured the extracting yield from *C. saiva* burs using four different extraction solvents including acetone, ethanol, methanol, and water, and found that the extraction yield decreased as the polarity of the solvent decreased.

The total polyphenols and tannins content in crude extract obtained using different solvents showed significant difference. As presented in Table 1, 50% ethanol extracts showed the highest total polyphenols and tannins content, followed by 75%, 30% ethanol and water extracts. This indicated that water extracts contained much more non-phenols or non-tannins, which would make it much more difficult for the following purification of polyphenols and tannin. With respect to total polyphenols yields from raw burs, 75% ethanol extraction was the lowest, and there was no significant difference between water, 30% ethanol and 50% ethanol extract. Meanwhile, the 50% ethanol extraction led to both highest total tannins content in extracts and highest yield of tannins from raw burs. Therefore, 50% ethanol was selected as the optimal solvent for extraction.

### 2.2. Isolation of Total Polyphenols and Tannins

To avoid the toxic organic solvents used in conventional separation techniques such as gel and high-speed counter-current chromatographic separation process and to increase the safety of the process, the adsorption and desorption on macroporous resins were utilized for isolation of chestnut bur polyphenols and tannins. In fact, macroporous resins have been successfully applied to the separation and isolation of effective components from many natural products, as an efficient method with a moderate purification effect, high absorption capacity, low operating costs, low solvent consumption, and easy regeneration [13,14,15]. After macroporous resin chromatography, the 50% ethanol extracts of chestnut burs were fractionated into two parts, Fraction I washed out with water and Fraction II eluted with 50% ethanol. As presented in Table 2, the polyphenols and tannins contents in Fraction II were approximately 2.5- and 4.6-fold higher than those in Fraction I, and 1.34- and 1.39-fold higher than those in crude extract.

Plant tannins constitute a complex group of naturally occurring polymers, and a rigorous chemical definition is difficult. Thus, tannins can conveniently be divided into two groups based on their structures, hydrolysable and condensed tannins [16]. To identify the predominant tannins in chestnut burs crude extracts and fractions, hydrolysable or condensed tannins, the content of condensed tannins was determined. As illustrated in Table 2, the content of condensed tannins was very low both in extracts and fractions, contributing less than 2% to the total tannins, which indicated that the predominant tannins in chestnut burs were hydrolysable tannins. This result was highly consistent with the report of Vázquez [10], who characterized the polyphenols of *C. sativa* burs as hydrolysable gallotannins based on MALDI-TOF spectrum.

Alasalvar [17] used Sephadex LH-20 chromatography to separate crude extracts of hazelnut skin into low-molecular-weight polyphenols (Fraction One) and tannins (Fraction Two) with 95% ethanol and 50% acetone as mobile phases, respectively. The total polyphenols content in crude extract, Fractions One and Two was 686 ± 7, 441 ± 12, and 697 ± 11 mg/g, respectively. This means the polyphenols contents in Fraction Two were approximately 1.58-fold higher than those in Fraction One, and 1.02-fold higher than those in crude extract. Therefore, the isolation of polyphenols and tannins using Sephadex LH-20 chromatography seemed less effective than the macroporous resin chromatography used in the present study. Ogawa [7] used Diaion HP-20, Chromatorex ODS 1024T, and Sephadex LH-20 chromatography to fractionate the seed shells of *Aesculus turbinate*, and highly purified polyphenols fractions were obtained. However, three kinds of chromatography were used including macroporous resin, normal-phase and reverse-phase chromatography, which was very complicated and too much toxic methanol was needed. Therefore, this strategy seems much more suitable for analysis than for industrial application. The one-step HPD-100 macroporous resin chromatography procedure established here provided an effective, low cost and simple method for chestnut industries to extract and purify polyphenols or tannins from Chinese chestnut spiny burs, which would facilitate the utilization of the resources of chestnut burs discarded as a waste conventionally.

### 2.3. Scavenging of DPPH and ABTS Radicals

**Figure 1:**
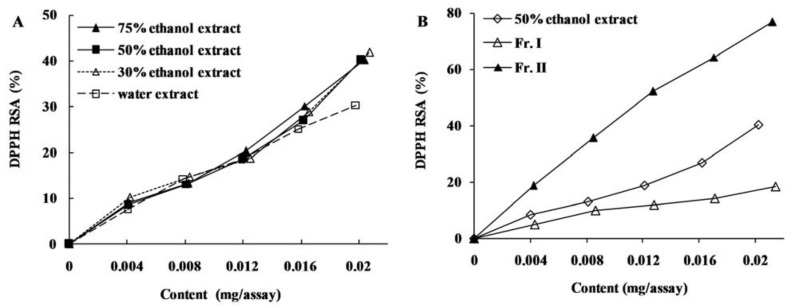
DPPH radical scavenging activity (RSA) of *C. mollissima* burs extracts (**A**) and fractions (**B**).

**Figure 2:**
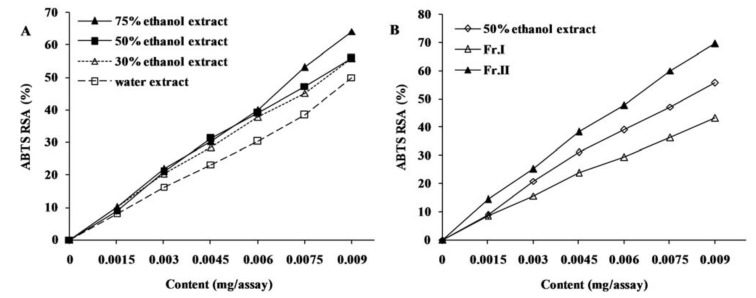
ABTS radical scavenging activity (RSA) of *C. mollissima* burs extracts (**A**) and fractions (**B**).

The DPPH and ABTS radical scavenging assays are usually employed to evaluate the ability of antioxidants to scavenge free radicals. As the reaction between antioxidant molecules and radicals progresses, the absorbance of the reaction system decreases. Hence, the change of absorbance is used as a measure for the scavenging of DPPH and ABTS^+^ radicals, and the more rapidly the absorbance changes, the more potential antioxidant activity the samples possess [17]. In the present study, all four different solvent extracts of chestnut burs exhibited considerable DPPH radical scavenging activities, and no significant difference was observed between 30%, 50% and 75% ethanol extracts (Figure 1A and Figure 2A). However, the DPPH and ABTS^+^ radical scavenging activity of water extract was lower than the others. Similar results were observed for EC_50_ values (Table 3), which are defined as micrograms of extracts or fractions per assay required for 50% scavenging of DPPH and ABTS^+^ radicals. For four kinds of extracts, 75% ethanol extract showed the lowest EC_50_, indicating it possesses the greatest antiradical activity, followed by 50% ethanol, 30% ethanol and water extracts. With respect to the fractions, Fraction II showed higher radicals scavenging activities than crude extract and Fraction I (Figure 1B and Figure 2B), which indicated that Fraction II was effectively enriched in constituents responsible for radicals scavenging activity after macroporous resin chromatography.

### 2.4. Reducing Power

**Figure 3:**
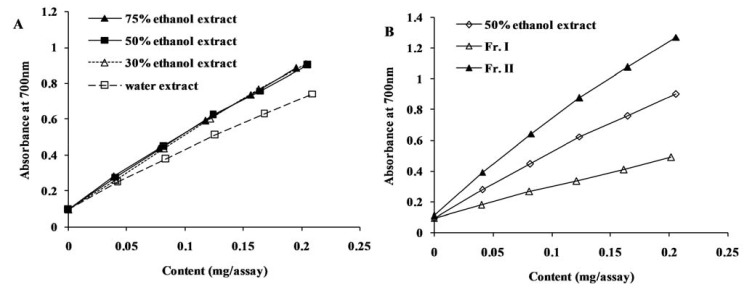
Reducing power of *C. mollissima* burs extracts (**A**) and fractions (**B**).

The reducing power of the crude extracts and their fractions of chestnuts burs is depicted in Figure 3. The absorbance of the reaction system was highly correlated with the concentration of extracts (R^2^ > 0.97), and the higher slope of the line indicated higher reducing power of the samples. In comparing the 30%, 50% and 75% ethanol extracts, the slope of water extracts was the smallest, and the slopes of 30%, 50% and 75% ethanol extracts were almost the same (*p* > 0.05). With respect to Fraction I and Fraction II, the slope of Fraction II was much greater than both the crude extract and Fraction I. The result of reducing power assay is highly consistent with that of DPPH and ABTS radical scavenging assay.

### 2.5. Cellular Antioxidant Activity

The cellular antioxidant activity (CAA) assay was developed by Liu to measure the antioxidant activity of antioxidants in cell culture in response to a need for a more biologically representative method than the chemical antioxidant activity assays as mentioned above including DPPH, ABTS radical scavenging assay and reducing power assay [17]. The CAAs of extracts and fractions of chestnut burs were measured with two protocols (PBS wash and no PBS wash), as described in literature [18]. The EC_50_, CAA values and median cytotoxicity doses are listed in Table 4.

In the no PBS wash protocol CAA assay, the 50% ethanol extraction had the highest CAA values and lowest EC_50_ values in the four kinds of extractions, followed by 75% ethanol, 30% ethanol and water extractions. However, the CAA values for four kinds of extracts were not significantly different from each other. In the PBS wash protocol, the same trend for CAA values was observed. With respect to EC_50_ in CAA assay with PBS wash protocol, the 75% ethanol extract showed the lowest values, followed by 50%, 30% ethanol and water extract, but no significant difference was observed between 50% and 75% ethanol extracts.

As illustrated in Table 4, in both PBS wash and no PBS wash protocol CAA assay, Fraction II exhibited significant higher CAA values and lower EC_50_ values than 50% ethanol extracts and Fraction I. These results indicated that macroporous resin chromatography significantly enhanced not only the chemical antioxidant activity, but also the cellular antioxidant activity of chestnut burs in Fraction II.

### 2.6. Correlation Analyses

Using regression analyses, the relationships between total phenolic content, total tannins content and EC_50_ of DPPH and ABTS radicals scavenging values, as well as CAA values were determined. Total polyphenols were significantly negatively correlated to EC_50_ values of DPPH radicals scavenging (R^2^ = 0.550, *p* < 0.05) and ABTS radicals scavenging (R^2^ = 0.764, *p* < 0.05). Total tannins content was more significantly negatively correlated to EC_50_ values of DPPH radicals scavenging (R^2^ = 0.598, *p* < 0.05), and ABTS radicals scavenging (R^2^ = 0.817, *p* < 0.05) than those of poly-phenols. Total polyphenols were significantly positively correlated to CAA values (R^2^ = 0.403, *p* < 0.05 for the no PBS wash protocol; R^2^ = 0.515, *p* < 0.05 for PBS wash protocol). Total tannins content was also significantly positively correlated to CAA values (R^2^ = 0.452, *p* < 0.05 for no PBS wash protocol; R^2^ = 0.512, *p* < 0.05 for PBS wash protocol). These correlation analyses revealed that the antioxidant activity of the chestnut burs extracts and fractions was highly correlated with the content of phenolic and tannins in the chestnut burs.

## 3. Experimental

### 3.1. Plant Samples and Reagents

The Chinese chestnut burs were harvested at a chestnut plantation in Qianxi, Hebei Province of China at the beginning of the harvest season of 2008, authenticated by Dr Yujun Liu, Beijing Forestry University. The burs were air-dried till equilibrium humidity, ground and transferred to darkness for further use. All chemicals were purchased from Sigma-Aldrich, Inc. unless otherwise specified. HepG2 human liver cancer cells were kindly offered by Professor Du Lijun, Tsinghua University, China.

### 3.2. Extraction of Chestnut Burs Polyphenols and Tannins

The chestnut burs powder was extracted using four different solvent systems [water, 30:70 (v/v) ethanol/water, 50:50 (v/v) ethanol/water and 75:25 (v/v) ethanol/water] at a solid to solvent ratio of 1:10 (w/v), and subsequently placed in a shaking constant-temperature water bath at 80 °C for 1 h. The resulting slurries were centrifuged at 5,000 g for 10 min to obtain the supernatant. The residues were subjected to repeated extraction twice under the same condition and the extracts supernatants were collected, combined and then evaporated under vacuum at 50 °C. After concentrated, the extract was lyophilized at −50 °C and 0.1 MPa, and finally restored at −20 °C in vacuum-sealed colored pouches for further analysis. The extraction yields are expressed by reference to dry matter.

### 3.3. Fractionation of Chestnut Bur Extracts

The chestnut burs were extracted with 50% ethanol according to the methods described above, and the supernatants were combined after centrifugation and evaporated till 20% volume left. Then, the extracts were replenished with distilled water to the initial volume and centrifuged at 5,000 g for 10 min to obtain the supernatant. Subsequently, the supernatant was subjected to a column chromatography (600 mm × 60 mm i.d.) packed with HPD 100 macroporous resin (Cangzhou Bonchem Co., Ltd, Cangzhou, China) and equilibrated with distilled water. Fraction I was eluted with 2,000 mL distilled water, and Fraction II was obtained after elution with 2,000 mL of 50% ethanol as mobile phase. Both Fraction I and Fraction II were evaporated, lyophilized and finally stored at −20 °C in darkness for further analysis.

### 3.4. Determination of the Content of Total Polyphenols, Total Tannins and Condensed Tannins 

The content of total polyphenols was estimated using the Folin-Ciocalteu’s phenol reagent as described by Amarowicz *et al.* [19]. The content of total tannins was determinate based on phosphomolybdium tungstic acid-casein reaction, described in Chinese Pharmacopoeia 2005 [20]. Briefly, sample solution (25 mL) was quickly mixed with casein (0.6 g) and incubated at 30 °C for 1 h. After filtration, the supernatant was collected. Then, the polyphenols content in original solution and supernatants were assessed using Folin-Ciocalteu’s phenol reagent, and the total tannins were defined as polyphenols which could be absorbed by casein. The total polyphenols and total tannins results were expressed as g GAE / 100 g extract (on a dried weight basis). The content of condensed tannins was determined according to a vanillin/HCl method [21], and the results were expressed as g CE (Catechin Equivalent) / 100 g extract (on a dried weight basis).

### 3.5. Determination of DPPH and ABTS Radical Scavenging Activity

The method described by Alasalvar [17] was used to assess DPPH and ABTS radicals scavenging activities of the extracts and fractions. For DPPH radicals assay, aqueous solutions (0.1 mL) of chestnut bur extracts or fractions with concentration of 40–200 μg/mL were mixed with a freshly prepared DPPH solution (0.25 mL, 1 mM in methanol) and ethanol (2 mL). The mixture was shaken vigorously and left to stand for 20 min in the dark at room temperature and the absorbance was read at 517 nm. For ABTS radicals assay, an ABTS solution (7 mM) was mixed with potassium persulfate (140 mM) with a ratio of 62.5:1 for 16 h in the darkness at room temperature to produce ABTS radical cation (ABTS^+^) stock solutions. The ABTS^+^ stock solution was diluted with ethanol to an absorbance of 0.70 at 734 nm as a working solution. An aliquot (0.15 mL) of aqueous solution containing 10 to 60 μg/mL of chestnut bur extract or fraction was mixed thoroughly with the ABTS^+^ solution (2.85 mL) and after 6–10 min in the darkness at room temperature; the absorbance was read at 734 nm. The radical-scavenging activity (RSA) for DPPH and ABTS radicals was calculated as %RSA = 100 (A_0_ − A_s_)/A_0_, where A_s_ is the absorbance of the extract solution and A_0_ is the absorbance of a control solution prepared without extracts.

### 3.6. Determination of Reducing Power

The method described by Oyaizu [22] was used to assess the reducing power of the extract and its fractions. Briefly, an aliquot (1 mL) of aqueous solution containing 0.04 to 0.2 mg extract or its fractions was mixed with 0.2 M phosphate buffer (2.5 mL, pH 6.6) and 1% (w/v) solution of potassium ferricyanide [K_3_Fe(CN)_6_] (2.5 mL). After incubation in water bath at 50 °C for 20 min, 10% (w/v) trichloroacetic acid solution (2.5 mL) was added, and then the mixture was incubated at room temperature for 10 min. Subsequently, the mixture (2.5 mL) was combined with distilled water (2.5 mL) and 0.1% (w/v) solution of ferric chloride (FeCl_3_) (0.5 mL). Finally, absorbance of the reaction mixture was recorded at 700 nm. Results were expressed as the concentration of extract or fractions per assay versus absorbance at 700 nm.

### 3.7. Determination of Cellular Antioxidant Activity

The cellular antioxidant activity (CAA) was determined using the protocol established by Liu *et al.* [18,23]. Briefly, complete medium (Williams’ Medium E supplemented with 5% fetal bovine serum, 10 mM Hepes, 2 mM L-glutamine, 5.0 μg/mL insulin, 0.05 μg/mL hydrocortisone, 50 units/mL penicillin, 50 μg/mL streptomycin, and 100 μg/mL gentamycin) was used as growth medium for human HepG2 liver cancer cells. Then, cells were seeded on a 96-well microplate at a density of 6 × 10^4^/well in 100 μL of growth medium/well. Twenty-four hours later, the growth medium was removed, and all wells were washed with phosphate buffered saline (PBS, 100 μL). After washing, wells were treated with 100 μL solvent control, quercetin control, or tested samples plus 25 μM DCFH-DA (2′,7′-dichlorofluorescin diacetate) for 60 min. Then cells were treated with Hanks’ Balanced Salt Solution (100 μL) with 10 mM Hepes and 600 μM 2.2′-azobis(2-amidinopropane) (ABAP). Finally, emission at 538 nm was measured after excitation at 485 nm with a Fluoroskan Ascent FL plant reader at 37 °C every 5 min for 60 min. The EC_50_ (median effective dose) was determinated for extracts and fractions as described in literature, and EC_50_ values were converted to CAA values expressed as micromoles of quercetin equivalents per 100 g extracts [24]. The cytotoxicity of each extraction or fraction was determinate as described in literatures [18,23].

### 3.8. Statistical Analysis

Values are presented as mean ± standard deviation (SD) (n = 3) for each extract and fractions. The statistical significance (*t*-test: two-sample equal variance, using two-tailed distribution) was determined using Microsoft Excel statistical software (Microsoft Office Excel 2007, Microsoft Corp. Redmond, WA, USA). Differences at *p* < 0.05 were considered to be significant.

## 4. Conclusions

The present study investigated the extraction and isolation process of chestnut burs tannins, as well as the tannins’ chemical and biological activity as natural antioxidants and antiradicals. An effective, low cost and simple procedure for tannin extraction and isolation using 50% ethanol as extraction solvent and one-step HPD-100 macroporous resin chromatography with 50% ethanol as elution solvent was established. The phenol and tannin enriched extracts obtained using this method exhibited considerable DPPH and ABTS radical scavenging activity, reducing power, and cellular antioxidant activity. The fraction responsible for antioxidant activities in Chinese chestnut burs was primarily characterized as hydrolysable tannins. To the best of our knowledge, this is the first report of enrichment of tannins from burs of *C. mollissim* using macroporous resin chromatography, and to assess their cellular antioxidant activity. This study extends our knowledge about the potential bioactivities and applications in cosmetic, pharmaceutical and food processing industries of tannins abundant in the chestnut burs.

## Figures and Tables

**Table 1:** Extracting yield and total polyphenols and tannins contents in chestnut burs extract.

Gallic Acid Equivalent (GAE)	Water	30% Ethanol	50% Ethanol	75% Ethanol
yield g / 100 g bur	16.28 ± 0.69 ^A^	13.60 ± 0.93 ^B^	11.67 ± 0.76 ^C^	6.62 ± 0.53 ^D^
Total polyphenols g GAE / 100 g Extract	43.75 ± 1.62 ^A^	48.20 ± 1.25 ^B^	60.00 ± 1.49 ^C^	50.00 ± 1.15 ^B^
Total polyphenols g GAE / 100 g bur	7.12 ± 0.89 ^A^	6.56 ± 0.76 ^A^	7.00 ± 1.02 ^A^	3.31 ± 0.62 ^B^
Total tannins g GAE / 100 g extract	27.00 ± 1.9 ^A^	28.40 ±1.09 ^A^	44.00 ± 1.34 ^B^	36.60 ± 1.26 ^C^
Total tannins g GAE / 100 g bur	4.39 ± 0.54 ^A^	3.86 ± 0.39 ^B^	5.13 ± 0.43 ^C^	2.42 ± 0.51 ^D^

^A,B,C,D^ In each line with different letters means significant differences (*p* < 0.05).

**Table 2:** Total polyphenols and tannins content in fractions.

	Crude extract	Fraction II	Fraction I
Total polyphenols (mg GAE / g)	600.0 ± 14.9 ^A^	801.4 ± 15.6 ^B^	318.9 ±18.9 ^C^
Total tannins (mg GAE / g)	440.0 ± 13.4 ^A^	611.5 ± 16.4 ^B^	132.2 ± 13.2 ^C^
Condensed tannins (mg CE / g)	9.00 ± 0.04 ^A^	11.50 ± 0.05 ^B^	2.70 ± 0.03 ^C^

GAE: Gallic Acid Equivalent; CE: Catechin Equivalent. In each line different letters mean significant differences (*p* < 0.05).

**Table 3:** Antiradical activities in chestnut burs extracts and fractions.

	EC_50_ (μg/assay)					
	Water extraction	30% Ethanol extraction	50% Ethanol extraction	75% Ethanol extraction	Fraction II	Fraction I
DPPH	50.9	36.0	35.9	33.8	11.4	188
ABTS	10.6	8.48	8.03	7.03	5.80	12.5

**Table 4:** Cellular antioxidant activity of chestnut burs extracts and fractions.

	no PBS wash		PBS wash		cytotoxicity
	EC_50_ (mg/mL)	CAA (μmol of QE/100g)	EC_50_ (mg/mL)	CAA (μmol of QE/100g)	CC_50_ (mg/Ml)
water extraction	38.7 ± 3.5 ^A^	11.6 ± 0.4 ^A^	389 ± 34 ^A^	1.86 ±0.29 ^A^	249.3
30% ethanol extraction	36.5 ± 2.9 ^A^	11.9 ± 0.6 ^A^	334 ± 22 ^B^	1.98 ± 0.26 ^A^	213.5
50% ethanol extraction	33.5 ± 3.1 ^A^	12.6 ± 0.5 ^A^	276 ± 19 ^C^	2.86 ± 0.23 ^B^	224.9
75% ethanol extraction	34.6 ± 3.3 ^A^	12.3 ± 0.3 ^A^	266 ± 12 ^C^	2.71 ± 0.15 ^B^	306.5
Fraction I	71.6 ± 4.1 ^B^	10.2 ± 0.4 ^B^	556 ± 48 ^D^	1.11 ± 0.11 ^C^	300.4
Fraction II	14.2 ± 2.2 ^C^	54.2 ± 2.6 ^C^	109 ± 12 ^E^	5.66 ±0.20 ^D^	278.5

QE: quercetin equivalent. In each column different letters mean significant differences (p < 0.05).
